# Psychiatric Comorbidity and Economic Hardship as Risk Factors for Intentional Self-Harm in Gambling Disorder—A Nationwide Register Study

**DOI:** 10.3389/fpsyt.2021.688285

**Published:** 2021-10-18

**Authors:** Anna Karlsson, Olivia Hedén, Helena Hansson, Jenny Sandgren, Anders Håkansson

**Affiliations:** ^1^Department of Clinical Sciences Lund, Psychiatry, Faculty of Medicine, Lund University, Lund, Sweden; ^2^Department of Pediatrics, Skåne University Hospital, Malmö, Sweden; ^3^Faculty of Social Sciences, School of Social Work, Lund University, Lund, Sweden; ^4^Clinical Studies Sweden – Forum South, Skåne University Hospital, Lund, Sweden; ^5^Malmö Addiction Center, Region Skåne, Malmö, Sweden

**Keywords:** gambling disorder (GD), suicidality, suicide attempt, self-harm, social welfare payments, criminality, risk factors, comorbidity

## Abstract

**Background:** There is an increased risk of suicidality in gambling disorder (GD) and economic hardship is common in the population. Economic hardship itself is a risk factor for suicidality. This study aims to explore the risk of intentional self-harm in GD utilizing social welfare payment (SWP) as a proxy for economic hardship and exploring how economic hardship, gender, criminality, socioeconomic-, and psychiatric risk factors might contribute to intentional self-harm in GD.

**Methods:** This is a nationwide register-based study of 848 individuals diagnosed with GD in the Swedish healthcare system during the years of 2011–2014 with an average follow up of 4.9 years. Pearson's Chi-square analyses were carried out for comparisons regarding psychiatric comorbidity and intentional self-harm with regards to gender and SWPs. Univariable and multivariable Cox regression were utilized to analyse risk factors for intentional self-harm.

**Results:** A large part of the study population received SWPs (45.5% with an insignificant overrepresentation of women) and psychiatric disorders were more common in these individuals (*p* < 0.001). Conviction for crime in general (*p* < 0.001) as well as intentional self-harm (*p* = 0.025) were also more common amongst recipients of SWPs. Criminal conviction in general was abundant (26.5%). In the stepwise multivariable regression, substance-related diagnoses as well as anxiety, depressive, and personality disorders remained risk factors for intentional self-harm and no significant results were found with regards to gender, criminal history, or SWPs.

**Conclusions:** Social welfare payment was common among GD patients and intentional self-harm was more common amongst recipients than GD patients as a whole. Social welfare payments were however not a significant risk factor for intentional self-harm. However, attention to suicidality and self-injurious behavior should be paid from social services controlling SWPs due to the large prevalence of intentional self-harm in this group. In accordance with previous studies, comorbid psychiatric disorders such as anxiety, depression, substance use, and personality disorders increased the risk of intentional self-harm.

## Introduction

Gambling disorder (GD) is an addictive disorder with an approximate prevalence of 0.5% (1, 2), however, the treatment seeking rate is estimated to be only around 10% and many remain undiagnosed (3). Suicidality and psychiatric comorbidity are common in GD (4–6), women more often being diagnosed with co-occurring psychiatric disorders in general and affective- and anxiety disorders in particular (7). Although suicide levels and suicide attempts in GD have been sparsely described in clinical nationwide samples, a prior study from our research group estimates a 1.8-fold increase in the general mortality and a 15-fold increase in suicide mortality (for which depression was the only significant risk factor) for individuals with GD (8). As a matter of fact, few studies have investigated the relation between GD and completed suicide. Disordered gambling has however been found in so called “psychological autopsies” investigating the lives of suicide victims prior to death (9).

Suicidality is a complicated concept in register research, often defined as suicidal thoughts and suicide attempts. Neither the International Classification of Disease System 10 (ICD 10) nor the Diagnostic and Statistical Manual of Mental Disorders 5 (DSM 5) can differ between acts of suicidal intent and of self-injury (10, 11). Further, no specific diagnosis describes suicidal thoughts (10, 11). Self-injury and self-harm are often associated with emotionally unstable personality disorder and can often be an attempt at anxiety relief but can occur in many other individuals as well (12). Self-harm is however an important risk factor for suicide (13, 14). A survey study from Connecticut concluded that self-injurious behavior was associated with at-risk and problem gambling in youth and is to our knowledge the only study investigating self-injurious behavior related to problem gambling (15).

Studies on suicidality and GD are however more abundant. A study from our research group indicate elevated levels of suicide attempts and acts of intentional self-harm on a national level, with one in five individuals with GD having attempted or completed an act of self-harm/suicide (16). Further, little is known about women with GD and suicide even though many studies argue that female gender is an independent risk factor of suicidality in GD (17–19).

It appears as if criminal activity is markedly more common in individuals with GD (20). Further, individuals committing gambling related crime appear to suffer from worse gambling addiction, however no difference has yet been detected with regards to suicidality (21).

Further, economical risk factors have been found to increase the risk of suicidality and suicide in general (22, 23), and indebtedness has been found to be related to suicidality in GD in particular (24). Economic hardship and indebtedness are common amongst individuals with GD (24, 25), and personal debt has been described as an aggravating factor in GD (25). There is to our knowledge very little research on the effects of financial hardship and indebtedness on suicidal behavior although a Singaporean hospital record study indicated an association between indebtedness and past suicide attempts (however self-reported) (26).

An Australian study concluded that gambling related debt was greater in men than in women (27). However, a Swedish study concluded that over-indebtedness was more abundant in female gamblers and hypothesized that the gender related income differences between men and women might contribute to women being more likely to experience over-indebtedness although gambling debts have been described to be larger among men (28).

Women might suffer greater economic disadvantages from gambling (28), are to a greater extent burdened by psychiatric comorbidity (2, 7, 29, 30), and more often gamble as a mean to escape negative emotions (31). Our research group considers these facts, together with the notion that gambling to a large extent is being perceived as part of the male norm (32), underlines the need for gender specific research as we hypothesize that women with GD experience greater guilt and stigmatization contributing to increased suicidality.

Social welfare payments (SWP) are in Sweden granted to those individuals or households that are not able to cover their regular, most basic, expenses on their own (33). Included here are amongst other costs expenses regarding food, clothing, housing, leisure activities, and health and hygiene (34), and other necessary expenses such as costs for dental or medical care and child care (33). In general, recipients of welfare benefits in the form of SWP are worse off regarding self-reported health, social relations, and experience of poverty and do to a lesser extent have lower educational achievements and employment levels than those who do not receive such aid (35–37). A recent case-control study on problem gamblers suggests that financial risk factors in addition to psychiatric risk factors might contribute to the risk of attempting suicide, and suicide attempters were four times as likely to have payment defaults (38).

The knowledge on self-harm in GD is not well-known and previous research based on registry data cannot separate between suicide attempts and intentional self-harm due to the nature of the diagnostic coding. However, based on studies examining completed suicide or death from intentional self-harm, it is fair to assume that the majority of those deceased passed away due to actual suicide. Since the aim of this study was not to assess risk factors for completed suicide, we chose to utilize the term intentional self-harm which could indeed be self-injurious behavior—or a suicide attempt with a non-lethal outcome.

The main aim of this study is thus to investigate risk factors for intentional self-harm in individuals with GD focusing on gender, socio-economic risk factors, primarily SWPs but also criminality in relation to previously known psychiatric risk factors.

## Methods

This is a national cohort study based on register data provided by The Swedish National Board of Health and Welfare, Statistics Sweden and The Swedish National Council for Crime Prevention which hold several national registers.

### Materials and Procedure

The cohort was created by identifying individuals diagnosed with GD in Swedish healthcare during the years of 2011–2014 in the Swedish National Patient Register (NPR). The register contains data from specialized healthcare including emergency—inpatient and outpatient care. The NPR has been proposed to have a positive predictive value ranging between 85 and 95% for various kinds of psychiatric and somatic diagnoses (39). As part of the NPR, the Hospital Discharge Register has since the year of 1987 had a complete national coverage and 99% of all somatic and psychiatric hospital discharges have been registered (39). However, the hospital-based outpatient care coverage rates have been estimated to be lower, around 80% in 2011 (39). The lower rates regarding outpatient care coverage have been postulated to be due to missing data from, for example, private caregivers (40). Subsequent data have suggested that, missing data on diagnoses have diminished to 4% in outpatient psychiatry (41). Psychiatric diagnoses were categorized according to the ICD 10 system. In this article we will use the terminology of intentional self-harm with regards to diagnoses describing non-lethal suicide attempts and self-injurious behavior since the ICD 10 cannot differentiate between the two (10).

Data on criminal convictions were collected from the register on individuals found guilty of crime provided by The Swedish National Council for Crime Prevention. This register has a very high accuracy with only 0.001% cases missing a personal identification number in a study on 205,846 violent convictions (42). Apart from looking at crime in general, we looked at violent crime, sexual crime, acquisitive crime, economic crime, and drug crime. See Appendix A in Supplementary Material for specification of these categories.

The study also included data from The Register for SWPs held by the Swedish National Board of Health and Welfare. The overall assessment of financial aid need among the applicants is reviewed by a municipal social worker, and it is based on a standardized evaluation form taking into account social factors, work and efforts on the labor market (43). All of the municipalities across the nation provide information on an individual level (44). Regarding the quality of the register, the coverage rate of SWP has been proposed to be high but the unreported or incorrectly registered cases in Sweden is unknown (44). The information provided contains the monetary value of the SWP as well as number of months on SWP, the latter variable containing no missing data in contrast to the monetary value which is why this variable was chosen for the calculations. We will utilize SWP as a proxy variable for personal economic hardship and will thus not primarily investigate personal indebtedness but rather a need for financial contributions to make ends meet. Information on marital status and number of children were provided by Statistics Sweden.

Finally, information has been collected from the Swedish Cause of Death Register (CDR). In the CDR, the main cause of death is based on the information provided from the death certificate and is generally determined by the treating physician or, as is the case with unclear deaths or deaths from injuries, deaths amongst individuals utilizing drugs or alcohol and unnatural deaths such as accidents and suicide, by the forensic examiner (45). In turn, 95% of all deaths by suicide are reported to CDR (45).

There are often several causes of death listed on each deceased individual in the CDR. Self-inflicted harm without fatal outcome, a variable which indeed could involve various intents—from anxiety-alleviation to suicidal intentions—as well as various degrees of harm, were defined as the outcome variable. We chose to separate events of undetermined intent (i.e., ICD-10 codes X40–X49 or Y10–Y34) (8). This constituted a methodological choice previously suggested as preferable due to strongly confounding background factors related to events of undetermined intent as opposed to clear suicide diagnoses (46). Due to the low numbers of completed suicide (*n* = 7) no separate analysis on suicide death was performed since the statistical power would be inadequate.

The study was censored on December the 31, 2017, indicating that no data was retrieved after this date and that study follow-up ended on this date.

### Participants

In total, 878 patients were found to have been diagnosed with GD (pathological gambling, F63.0 according to the ICD-10, the diagnostic manual in use during the study period). One of these individuals was disqualified due to an erroneous personal identification number. Patients who were 18 years of age, or older, were included (*n* = 848) but minors were not. This was based on the assumption that receiving a GD-diagnosis as a minor might instead represent having a gaming disorder (8).

### Statistical Analysis

SPSS Statistics version 26 was used for carrying out statistical analyses and for compilation of descriptive data. Age distribution was presented in median and interquartile ranges (IQR). Follow up time was analyzed in order to evaluate the robustness of the data considering the fact that GD is being increasingly recognized in the Swedish health care system.

Pearson Chi-square analyses were conducted to investigate possible gender differences as well as differences between recipients of SWP and non-recipients with regards to psychiatric and socioeconomic factors.

As for the gender comparison, differences in SWP, acts of self-harm, psychiatric comorbidities, and substance use were investigated. This analysis was carried out to investigate potential differences between men and women at a group level in their overall mental health and socioeconomic situation, as we hypothesized that psychiatric comorbidity and need for SWP would be greater amongst women.

The comparative analysis on individuals receiving SWP and not receiving SWP included factors such as psychiatric comorbidity, criminality, and intentional self-harm factors which might be of potential interest to social services already involved with those individuals receiving SWP.

A separate analysis was also conducted comparing prevalence of crime committed during follow up investigating differences in drunk driving, violent-, sexual-, property-, drug-, and economic crime.

In the comparative analysis between those with female and male gender a Bonferroni-Holm procedure was utilized to diminish the risk for type 1 error, due to the large number of tests analyzed. We chose to divide the variables according in four groups: socioeconomic factors (*n* = 1), self-harm (*n* = 4), substance use disorders (SUD) (*n* = 11), and psychiatric comorbidities (*n* = 8). For each of these groups, the Bonferroni-Holm procedure was used to test the significant results (with the significance level of alfa <0.005). Further a chi-square analysis was conducted comparing gender differences in criminal convictions.

Finally, simple multiple factor Cox regression analyses were assessed to investigate known, and hypothesized, risk factors of intentional self-harm, from which the individual had not passed away (i.e., ICD codes X60–X84 in the NPR and not the CDR). Criminality in general was also included due to the fact that this potential risk-factor has not been studied much in the GD context but is a known risk factor for suicidality in general (47). Further, previously known factors associated with suicidality or self-harm were included, i.e., gender (16), substance (16), and alcohol use disorders (AUD) (16), as well as psychiatric comorbid disorders such as psychotic disorder (48), personality disorder such as borderline personality disorder and narcissistic personality disorder (49), bipolar disorder (50), depressive disorder (16), and anxiety disorder (16). Results were presented as hazard ratios (HR) and described with a 95% confidence interval. Again, the Bonferroni-Holm procedure was used to diminish the risk of type 1 error (with the significance level of alfa <0.005). A multiple Cox regression analysis was then based upon remaining significant results from the single factor analysis. Results were considered significant if the *p*-value was <0.05.

### Ethical Considerations

The study procedures were carried out in accordance with the Declaration of Helsinki. However, the requirement for informed consent was waived because the study was based on administrative population-based registers. Information to the public about the ongoing project with contact information to the research group was launched at the Lund University platform “LUPOP” on 02052019 (51). The study was approved by the regional ethics committee of Lund, Sweden (file number: 2018/3).

## Results

The population consisted of 848 individuals, amongst whom 169 individuals were of female gender (19.9%). The average age at baseline was 38.23 years (median 37; IQR: 28–47) with a range between 18 and 84 years. The average time in study was 4.9 years (median 4.86; IQR: 3.83–6.09). Among the subjects who died during the observation time (*n* = 26, nine women and 17 men) the average time in study was 3.32 years (median 3.22; IQR: 1.19–5.05). Seven of these individuals (all men) passed away due to suicide (ICD 10 codes X60–X84) and two additional individuals passed away due to potential suicide (Y10–Y34). Together, these individuals had an average time in study of 3.07 years (median 3.30; IQR: 0.86–4.96).

The majority of the sample did not have any children (68.7%, men: 70.4%, women 62.1%) with men, being less likely to be parents [Pearson Chi square test: χ${}_{\left(6,838\right)}^{2}$ =13.3, *p* = 0.039]. The majority were non-married with 13% being married [12.8% of the men and 13.6% of the women, Pearson Chi square test: χ${}_{\left(1,848\right)}^{2}$ =14.6, 6 *p* < 0.001]. One-fifth were divorced (21.1%).

Out of all the study individuals, 731 individuals (86.2%) had concomitant psychiatric disorders (all ICD 10 “F-diagnosis” except F63.0, GD) (Table 1). In our population, 45.6% (*n* = 387) had been in both inpatient and outpatient psychiatric care. Of these individuals, 99.5% (*n* = 385) had psychiatric diagnoses co-occurring with GD. A total of 421 subjects (49.6%) had solely been in outpatient care during the observation time, and 74.8% of them had co-occurring psychiatric diagnoses. In total, a number of 107 (12.6%) subjects were found to have attempted or committed suicide or had made an act of intentional self-harm (X60–X84).

**Table 1:** Prevalence of psychiatric disorders, self-harm, and suicide.

**Selected variable (ICD 10 code)**	**Male gender**	**Female gender**	**Total (*n*, %)**	***p***	**Bonferroni-Holm significance level**
Social welfare payment	*N* = 301 (44.3%)	*N* = 85 (50.3%)	*N* = 386 (45.5%)	0.163	NA
Death from suicide X60–X84	*N* = 7 (1%)	*N* = 0 (0%)	*N* = 7 (0.8%)	1.000^**^	NA
Death from self-harm of unknown intent Y10–Y34	*N* = 2 (0.3%)	*N* = 0 (0%)	*N* = 2 (0.2%)	1.000^***^	NA
Non-lethal intentional self-harm X60–X84^*^	*N* = 71 (10.5%)	*N* = 31 (18.3%)	*N* = 102 (12%)	0.005	0.013
Non-lethal self-harm of unknown intent Y10–Y34	*N* = 16 (2.4%)	6 (3.6%)	22 (2.6%)	0.415^**^	NA
SUD F11–F19	*N* = 154 (22.7%)	*N* = 41 (24.3%)	195 (23.0%)	0.662	NA
Alcohol, F10	*N* = 188 (27.7%)	45 (26.6%)	233 (27.5%)	0.782	NA
Opioids, F11	*N* = 21 (3.1%)	6 (3.6%)	27 (3.2%)	0.762	NA
Cannabinoids, F12	*N* = 34 (5.0%)	7 (4.1%)	41 (4.8%)	0.639	NA
Sedatives, F13	*N* = 39 (5.7%)	18 (10.7%)	57 (6.7%)	0.023	0.005
Cocaine, F14	*N* = 5 (0.7%)	1 (0.6%)	6 (0.7%)	1.000^**^	NA
Other stimulants, F15	*N* = 18 (2.7%)	1 (0.6%)	19 (2.2)	0.146^*^	NA
Hallucinogens, F16	*N* = 4 (0.6%)	2 (1.2%)	6 (0.7%)	0.343^**^	NA
Tobacco, F17	*N* = 17 (2.5%)	7 (4.1%)	24 (2.8%)	0.296^*^	NA
Volatile solvents, F18	*N* = 1 (0.1%)	0 (0.0%)	1 (0.1%)	1.000^**^	NA
Multiple drug use and psychoactives, F19	*N* = 88 (13%)	24 (14.2%)	112 (13.2%)	0.670	NA
Psychiatric comorbidity *F* diagnosis (exluding F63.0)^*^	*N* = 572 (84.2%)	*N* = 159 (94.1%)	*N* = 731 (86.2%)	<0.001	0.001
Bipolar disorder F30–F31^*^	*N* = 69 (10.2%)	*N* = 35 (20.7%)	*N* = 104 (12.3%)	<0.001	0.006
Anxiety disorder F40–F48^*^	*N* = 352 (51.0%)	*N* = 113 (66.9%)	*N* = 465 (54.8%)	<0.001	0.007
Depressive disorders F32–F33^*^	*N* = 280 (41.2%)	*N* = 370 (43.6%)	*N* = 379 (43.6%)	0.005	0.013
Other depressive disorder F34–F39	*N* = 23 (3.4%)	*N* = 9 (5.3%)	32 (3.8%)	0.237	NA
Psychotic disorder F20–F29	*N* = 61 (9.0%)	*N* = 7 (4.1%)	*N* = 68 (8.0%)	0.038	0.017
Personality disorder, F60–F62 excluding antiscoial personality disorder (F602)^*^	*N* = 80 (11.8%)	*N* = 53 (31.4%)	*N* = 133 (15.7%)	<0.001	0.008
Antisocial personality disorder F602	*N* = 13 (1.9%)	*N* = 1 (0.6%)	*N* = 15 (1.7%)	0.227	NA

*Gender comparison through Pearson Chi square test and Fisher's test when needed. Significant results at p level 0.05 were then tested with the Bonferroni-Holm procedure dividing the factors into four groups; socioeconomic factors (n = 1), self-harm (n = 4), substance use disorders (n = 11), and psychiatric comorbidities (n = 8) with an alfa level at 0.05. N = 848. AUD, alcohol use disorder; SUD, substance abuse disorder*.

* *Significant at the alfa level of 0.05 after Bonferroni-Holm correction*.

*NA, not applicable*.

** *One of the cells (25%) had expected count <5. Results are thus from Fisher's test, two-sided results displayed*.

*** *Two of the cells (50%) had expected count <5. Results are thus from Fisher's test, two-sided results displayed*.

### Social Welfare Payments

Out of all subjects, 45.5% (*n* = 386, 85 women and 301 men) had received SWP at some point during the observation time. Amongst the women 50.3% received SWP and 44.3% of the men. The total number of months on such aid ranged between 1 and 84 months. The average (median) number of months on aid was 9.0 months (3.0–28.0) and the mean number of months on SWP was 17.8 months with a standard deviation of 19.6 months. The average (median) time in study among individuals on SWP was 5.07 (3.94–6.31) years.

Significant differences were found between those with and without SWP regarding psychiatric disorders in general [χ^2^(1, 848) = 21.6, *p* < 0.001] and depressive disorder [χ^2^(1, 848) = 4.11, *p* = 0.043], anxiety disorder [χ^2^(1, 848) = 32.8, *p* < 0.001], personality disorder [χ^2^(1, 848) = 19.5, *p* < 0.001], AUD [χ^2^(1, 848) = 4.6, *p* = 0.031], and SUD [χ^2^(1, 848)=39.3, *p* < 0.001] all of which were more common among recipients of SWP. Conviction for crime in general [χ^2^(1, 848) =24.3, *p* < 0.001] as well as non-lethal self-harm (X60–X84), [χ^2^(1, 848) = 4.1, *p* = 0.042] were also more common amongst those who had received SWP (Table 2).

**Table 2:** Prevalence of psychiatric comorbidity amongst recipients of social welfare payment compared with Pearson Chi square test to non-social welfare payment recipients.

**Selected variable (ICD 10 code)**	**No social welfare payment**	**Social welfare payment**	***p***
	**(*n*/%)**	**(*n*/%)**	
AUD^*^	113 (24.6%)	120 (31.1%)	0.031
SUD^*^	68 (14.7)	127 (32.9)	<0.001
Psychiatric comorbidity^*^	375 (81.2)	356 (92.9)	<0.001
Bipolar disorder, F30–F31	53 (11.5)	51 (13.2)	0.442
Anxiety disorder. F40–F48^*^	212 (45.9)	253 (65.5)	<0.001
Depressive disorder, F32–F33^*^	187 (40.5)	183 (47.4)	0.043
Personality disorders, F60–F62 exkluding antiscoial personality disorder (F602)^*^	48 (10.4)	85 (22.0)	<0.001
Psychotic disorders, F20–F29	33 (7.1)	35 (9.1)	0.304
Criminality^*^	91 (19.7)	134 (34.7)	<0.001
Non-lethal intentional self-harm (X60–X84)^*^	46 (10.0)	56 (14.5)	0.042

*N = 848. AUD, alcohol use disorder; SUD, substance abuse disorder*.

* * p <0.05*.

### Gender

Among study subjects, women were more likely to suffer from concomitant psychiatric disorders [χ${}_{\left(1,848\right)}^{2}$ =11.0, *p* < 0.001] (Table 1). Women were slightly more often recipients of SWP (50.3% compared to 44.3% of the men although not statistically significant) [χ${}_{\left(1,848\right)}^{2}$ =1.9, *p* = 0.163] (Table 1).

Of the seven certain suicides (diagnoses X64–X80 in the CDR) [χ${}_{\left(1,848\right)}^{2}$ =1.7, *p* = 0.185] all had been committed by men; however, the difference did not reach statistical significance (Table 1). As many as 18.3% of the women and 10.5% of male study subjects had an episode of intentional self-harm [χ${}_{\left(1,848\right)}^{2}$ =7.9, *p* = 0.005] (Table 1).

### Criminality

Roughly one in four study subjects (26.5%) had been sentenced for a criminal conviction during follow up. This was more common in men although one in five women had also received a sentence [χ${}_{\left(1,848\right)}^{2}$ = 8.4, *p*= 0.004]. Property crime appeared to be the most common amongst the women whilst the men had committed a larger variety of criminal acts (Table 3). Further, criminal conviction was more common in recipients of SWP [χ${}_{\left(1,848\right)}^{2}$ = 24.3, *p* < 0.001] (Table 2).

**Table 3:** Prevalence of criminal conviction during follow up.

**Category of crime**	**Total**	**Men**	**Women**	***P*-value**
	***n* (%)**	***n* (%)**	***n* (%)**	
Violent crime	40 (7.7)	36 (5.3)	4 (2.4)	0.107
Sexual crime	3 (0.35)	3 (0.4)	0 (0)	0.387
Property crime	61 (7.2)	51 (7.5)	10 (5.9)	0.473
Drunk driving	26 (3.1)	21 (3.1)	5 (3.0)	0.928
Drug crime	42 (5.0)	38 (5.6)	4 (2.4)	0.083
Economic crime	65 (7.7)	58 (8.5)	7 (4.1)	0.054
Other crime^*^	67 (7.9)	60 (8.8)	7 (4.1)	0.043
Any crime^*^	225 (26.5)	195 (28.7)	30 (17.8)	0.04

*Gender comparison through Pearson Chi square test. N = 848*.

* *Significant at the alfa level of 0.05 after Bonferroni-Holm correction*.

### Risk Factors for Intentional Self-Harm

The non-adjusted, single factor Cox regression analyses showed that SWP was not statistically significantly associated with intentional self-harm. However, criminal conviction, female gender, and psychiatric disorders such as AUD and SUD, personality disorders, and anxiety-, depressive-, and psychotic diagnosis appeared to be associated with intentional self-harm. Although when investigating the results with the Bonferroni-Holm procedure, female gender and criminal conviction did not reach statistical significance (Table 4).

**Table 4:** Univariable Cox regression investigating intentional self-harm (ICD 10 codes X60–X84).

**Risk factor (ICD 10 code)**	**Intentional self-harm (X60–X84)**	**Single factor cox regression**			**Bonferroni-Holm significance level**
	***n* (%)**	**HR**	**95% CI**	***p***	
Social welfare payment	56 (14.5)	1.443	0.977–2.132	0.066	NA
No social welfare payment	46 (10.0)				
Female gender	31 (18.3)	1.807	1.185–7.755	0.006	0.01
Male gender	71 (10.5)				
Age		1.000	0.984–1.016	0.996	NA
AUD (F10)^*^	46 (19.7)	2.214	1.499–3.270	<0.001	0.005
No AUD	56 (9.1)				
SUD (F11–F19) ^*^	52 (26.7)	3.648	2.473–5.382	<0.001	0.006
No SUD	50 (7.7)				
Psychotic disorder (F20–F29)	12 (17.6)	1.461	0.799–2.670	0.218	NA
No psychotic disorder	90 (11.5)				
Anxiety disorder (F40–48)^*^	81 (17.4)	3.262	2.019–5.272	<0.001	0.006
no anxiety disorder	21 (5.5)				
Depressive disorder (F32–33)^*^	72 (19.5)	3.229	2.109–4.943	<0.001	0.007
No depressive disorder	30 (6.3)				
Personality disorder (F60–62)^*^	37 (26.6)	3.055	2.039–4.577	<0.001	0.008
No personality disorder	65 (9.2)				
Bipolar disorder (F30–31)	17 (16.3)	1.435	0.852–2.415	0.174	NA
No bipolar disorder	85 (11.4)				
Criminal conviction	38 (16.9)	1.581	1.058–2.363	0.025	NA
No criminal conviction	64 (10.3)				

*Censoring (time of death, final emigration, or end of study: 20171231). Significant results at p level 0.05 were then tested with the Bonferroni-Holm procedure (n = 11). N = 848*.

* *Significant result at the alfa level 0.05 after Bonferroni-Holm procedure. AUD, alcohol use diagnosis; SUD, substance use diagnosis*.

*NA, not applicable*.

In the multivariable Cox regression analysis, the predictive value of bipolar disorder and alcohol use diagnosis (AUD), respectively, were no longer statistically significant for self-injury. However, substance use diagnosis (SUD) as well as anxiety disorder (HR: 1.920 95% CI:1.156–3.189, *p* = 0.012) depressive disorders (HR:2.385, 95% CI:1.539–3.695, *p* < 0.001), and personality disorders (HR: 2.195, 95% CI:1.446–3.331 *p* < 0.001) remained risk factors for intentional self-harm (Table 5).

**Table 5:** Multivariable Cox regression investigating intentional self-harm, from which the individual did not decease.

**Risk factor (ICD 10 code)**	**Multiple factor Cox regression**		
	**HR**	**95% CI**	***p***
AUD (F10)	1.419	0.930–2.164	0.104
SUD (F11–F19)^*^	2.404	1.569–3.683	<0.001
Anxiety disorder (F40–48)^*^	1.920	1.156–3.189	0.012
Depressive disorder (F32–33)^*^	2.385	1.539–3.695	<0.001
Personality disorder (F60–62)^*^	2.195	1.446–3.331	<0.001

*(ICD 10 codes X60–X85). Censoring (time of death, final emigration, or end of study: 20171231). N = 848. AUD, alcohol use diagnosis; SUD, substance use diagnosis*.

* * p <0.05*.

## Discussion

Although intentional self-harm was more abundant in women and in recipients of SWP previously known psychiatric risk factors i.e., substance related diagnosis, anxiety-, depressive-, and personality disorders were the factors which predicted intentional self-harm in the multivariable Cox regression. This does not mean that female gender and SWP are not per se risk factors for intentional self-harm, larger studies are needed to further analyse these relationships. It is however of clinical importance that psychiatric risk factors appear to be the strongest predictors of intentional self-harm and that there is need to assess GD in relation to psychiatric comorbidity.

Previous research has also found intentional self-harm and suicidality to be more frequent among women (16, 18, 52). The increased risk of suicide attempts among women have been proposed to be explained by higher prevalence of depression in the same group (53). Nevertheless, more recent studies have suggested that this risk was associated with female gender, also when controlling for depression (16, 17). It is apparent that the relationship between gender, psychiatric, and socioeconomic risk factors are complex in terms of suicidality and acts of self-harm.

The results from this study show that almost half of the population had received SWP during follow up. In comparison, recent data from the Swedish National Board of Health and Welfare show that around one in twenty households in the overall population were recipients of SWP in 2018 (33). Although the median follow-up in our study was close to 5 years, the results indicate a greater need for economic support for those with GD.

Future studies are needed to get a more comprehensive picture of indebtedness, economic hardship, and suicidality in GD. Accordingly, larger samples and perhaps other variables than SWP such as factors related to indebtedness might be better predictors of suicidality. In fact a recent study indicates that as many as 50% of problem gamblers had borrowed money for gambling and that these loans most often were from private acquaintances, followed by payday loans or “cash advances” and unsecured bank loans (54). Finding one single indicator for personal indebtedness or economic hardship might thus be hard.

We found no significant gender differences regarding prevalence rates of concomitant SUDs or AUD in our population in accordance with previous literature (7). Moreover, from the adjusted regression analysis in our study, SUD (excluding AUD) was found to be associated with an increased risk of intentional self-harm. The impact of SUD on suicide attempts have been described as three-fold, in previous literature (55). In addition, we found that psychiatric comorbidity including depressive-, anxiety-, bipolar-, and personality disorders were more common among women. This has also been demonstrated in previous studies (2, 7, 29, 30). Psychotic disorder however was more common amongst the male study participants. As expected, criminality in general was more common in men but surprisingly common amongst the women. Almost 18% of the women had been sentenced to a crime during follow up which is in line with previous findings from New Zeeland where Abbot et al., describe high rates of gambling problems amongst female prison clients (56).

Rates of criminality were overall high in the study sample, women most often being involved in acquisitive crimes whilst the male population had been sentenced to a variety of criminal categories such as violent crime, property crime, economic crime and “other criminal categories.” These results need further investigation beyond the scope of the present study. All in all, as many as one in four were sentenced to crime during follow up with a male dominance.

## Limitations

Although the register material was quite extensive and of high quality in this study, there are inevitable limitations to conducting a register study on GD patients. Indeed, we were only able to include individuals who had been in the medical care system. This constitutes a limitation because, in contrast to the burden of harm associated with GD and the need of effective interventions, it has been described that overall treatment-seeking is sparse (57, 58). Associations between overall help-seeking motivation and a lower degree of severity have been found (59). This could indicate that our study subjects might have had milder forms of the condition. However, this is rather unlikely considering the high rate of psychiatric comorbidity in our population, which instead is associated with more severe forms of the condition (60). As previously discussed, the inability to distinguish between self-injurious behavior in the diagnostic systems implicate that no conclusions on the suicidal intent of the self-harm can be drawn. This distinction can in certain cases be hard to make even for the psychiatric clinician and it is well known that women are over-represented in studies focusing on suicide attempts whilst the inverse is true for death from suicide.

Further, the percentage of women in the material make statistical conclusions hard to investigate and the gender comparisons are to be taken as rather rough estimations which need to be confirmed in larger studies. Although statistical power was weak in these analyses, we chose to present results with regards to potential gender differences as we very much believe that differences in comorbid diseases and life circumstances in individuals with GD are quite substantial and that attention must be paid to this in order to not draw erroneous conclusions for the group as a whole. Research on self-harm and suicidality in women with GD is much needed.

## Conclusion

This study confirms the notion that individuals with GD often are economically burdened, indeed, SWPs were common and utilized by approximately half of the population. Self-harm and suicidality were as previously described abundant. Further, criminal activity was common in the population with as many as one in four having been sentenced to crime during follow up. Risk factors for suicide attempt and self-harm were however several psychiatric diagnoses, i.e., SUD, anxiety disorders, depressive disorders, and personality disorder. It is evident that individuals with GD are in need of acknowledgment and aid from health care sectors, the prison and probation system and social services.

## Data Availability Statement

The datasets presented in this article cannot be readily available without the consent from the authorities holding the registers (i.e., The Swedish National Board of Health and The Swedish National Council for Crime Prevention). Inquiries regarding this procedure can be directed to Anna Karlsson, anna.karlsson.5043@med.lu.se.

## Ethics Statement

The studies involving human participants were reviewed and approved by The Regional Ethics Committee of Lund, Sweden (file number: 2018/3). The ethics committee waived the requirement of written informed consent for participation.

## Author Contributions

AK and AH designed and applied for ethical permission of the study. AK carried out the main part of the statistical analyses and wrote the first draft of the paper. OH contributed to statistical analysis and in writing of the paper. JS is a professional statistician who prepared the data upon which the analyses were run. AH and HH were involved during the process of designing and in writing of the paper. All authors made substantial contributions to the extension and finish of the paper, are responsible of the overall research idea and the background data and are equally responsible of the interpretation of data.

## Funding

This research was funded by the Swedish Southern Health Care Region Research (grant 2020-0424, Gambling Disorder—associations with suicidality, economic vulnerability, and mortality) and from AB Svenska Spel, the Swedish state-owned gambling operator (grant FO 2019-0013 Gambling disorder—associations with psychosocial problems, suicide, and crime).

## Conflict of Interest

AH holds a position as professor at Lund University financed in collaboration between Lund University and the Swedish gambling operator monopoly, Svenska spel AB, as a part of the latter part's responsibility for gambling and research policy. AK has received a grant from the same gambling operator monopoly, Svenska Spel AB as part of Svenska Spel ABs's responsibility for gambling research. The remaining authors declare that the research was conducted in the absence of any commercial or financial relationships that could be construed as a potential conflict of interest.

## Publisher's Note

All claims expressed in this article are solely those of the authors and do not necessarily represent those of their affiliated organizations, or those of the publisher, the editors and the reviewers. Any product that may be evaluated in this article, or claim that may be made by its manufacturer, is not guaranteed or endorsed by the publisher.
