# Supplementary material for: Psychiatric Comorbidity and Economic Hardship as Risk Factors for Intentional Self-Harm in Gambling Disorder—A Nationwide Register Study
Source: Front Psychiatry. 2021 Oct 18;12:688285. doi: 10.3389/fpsyt.2021.688285 (PMC8558368; doi:10.3389/fpsyt.2021.688285)
Supplement: Supplementary file 1 [file Data_Sheet_1.docx]

**Appendix A – Criminal Categories**

Criminal categories were derived utilizing statistics from the national criminal records registry held by The Swedish National Council for Crime Prevention. Seven different categories were created: violent crime, sexual crime, property crime, drug crime, economic crime, drunk driving and a category containing other crime. The register contains information on court verdicts, orders of summary punishment and a form of “failure to prosecute” which in Sweden implies that there is no doubt that the suspect has committed the crime, but it will not be brought up in court due to circumstances such as the crime being irrelevant in comparison to the other crimes for which the individual faces charges.

Categories of crime were determined after investigating previous studies utilizing Swedish criminal statistics and through examining the Swedish law and including crimes in relevant categories as well as investigating the categories of crime defined by the Swedish National Crime Council. We chose an approach where violent sexual crimes would be included in both the category ‘Sexual crime’ as well as ‘violent crime’.

Below is a list of specific laws utilized to create the categories in Swedish.

**ECONOMIC CRIME: EKONOMISK BROTTSLIGHET, BIDRAGSBROTTSLIGHET & BEDRÄGERI**

Bidragsbrottslagen (2007:612)

BRB 9 kap. Bedrägeri och annan oredlighet

BRB 10 kap. Förskingring, annan trolöshet och mutbrott:

BRB 11 kap. Borgenärsbrott

Skattebrott brott mot skattebrottslagen

Brott mot insiderlagen (Lag om straff för marknadsmissbruk på värdepappersmarknaden f.om 1 feb 2017, dessförinnan lagen om straff för marknadsmissbruk vid handel med finansiella instrument (2005:377)

Aktiebolagslagen

Lagen om näringsförbud (1986:436 Lag om näringsförbud, Lagen upphörde att gälla 2 augusti 2014, då lagen (2014:836) om näringsförbud trädde i kraft.

**DRUG CRIME: NARKOTIKABROTT**

Narkotikastrafflagen (1968:64)

Lag om straff för smuggling (2000:1225) (obs endast 6 §)

Narkotikasmuggling (6 §)

**DRUNK DRIVING: RATTFYLLERI**

Brott mot trafikbrottslagen (1951:649)

Obs endast paragraf 4, rattfylleri (alkohol & narkotika) och grovt rattfylleri (4a §)

**SEXUAL CRIME: SEXUALBROTT**
BRB 6 kap. Sexualbrott

(8 Redovisningen avser brott såväl mot den nya sexualbrottslagstiftningen (som trädde i kraft den 1 april 2005) som mot tidigare gällande lagstiftning.)

Våldtäkt (1 §)

Grov våldtäkt (1 §)

Sexuellt tvång (2 §)

"Sexuellt utnyttjande av person i beroendeställning (3 §) (Inklusive den tidigare lydelsen sexuellt utnyttjande resp. grovt sexuellt utnyttjande (3 §).

Våldtäkt mot barn (4 §) (Inklusive den tidigare lydelsen sexuellt utnyttjande resp. grovt sexuellt utnyttjande av underåriga (4 §).)

Grov våldtäkt mot barn (4 §)( 11 Inklusive den tidigare lydelsen sexuellt utnyttjande resp. grovt sexuellt utnyttjande av underåriga (4 §).)

Sexuellt utnyttjande av barn (5 §)

Sexuellt övergrepp mot barn (6 §) (12 Inklusive den tidigare lydelsen sexuellt umgänge med barn (6 §).)

Samlag med avkomling/syskon (7 §)( 13 Inklusive den tidigare lydelsen sexuellt umgänge med avkomling resp. med syskon (6 §).)

Utnyttjande av barn för sexuell posering (8 §)

Köp av sexuell handling av barn (9 §)1 14(Inklusive den tidigare lydelsen förförelse av ungdom (10 §).)

Kontakt med barn i sexuellt syfte (10a §) ( 15 Paragrafen trädde i kraft 1 juli 2009.)

Sexuellt ofredande (10 §) (16 Inklusive den tidigare lydelsen sexuellt ofredande (7 §).)

Köp av sexuell tjänst (11 §) 17 Paragrafen trädde i kraft 1 april 2005. Redovisningen kan även omfatta brott mot lagen (1998:408) om förbud mot köp av sexuella tjänster.

Koppleri (12 §)(18 Inklusive de tidigare lydelserna koppleri (8 §) och grovt koppleri (9 §).)

Grovt koppleri (12 §)

**PROPERTY CRIME: TILLGREPPSBROTT**

BRB
8 kap. Tillgreppsbrott

Stöld (1 §)

Ringa stöld (2 §)19

Grov stöld (4 §)

Tillgrepp av fortskaffningsmedel (7 §)

Grovt tillgrepp av fortskaffningsmedel (7 §)

Egenmäktigt förfarande (8 §)

Självtäkt (9 §)

Olovlig energiavledning (10 §)20

Brott mot 8 kap. (11 §)

Brott mot 8 kap. (12 §) För försök eller förberedelse till stöld, grov stöld, rån, grovt rån, tillgrepp av fortskaffningsmedel, grovt tillgrepp av fortskaffningsmedel, olovlig energiavledning eller grov olovlig energiavledning

Brott mot 8 kap. (13 §) Har annat i detta kapitel angivet brott än grov stöld, rån eller grovt rån förövats mot

1. någon som inte endast tillfälligt sammanbodde med gärningsmannen,

2. make, den som är i rätt upp- eller nedstigande släktskap eller svågerlag, syskon, svåger eller svägerska eller

3. någon annan som på liknande sätt är närstående till gärningsmannen, får åklagaren väcka åtal endast om målsäganden har angett brottet till åtal eller åtal är påkallat ur allmän synpunkt.

**VIOLENT CRIME: VÅLDSBROTT**BRB

3 kap. Brott mot liv och hälsa:

Fullbordat mord, dråp eller misshandel med dödlig utgång

Misshandel inkl. grov

- därav mot barn 0-6 år
- mot barn 7-14 år
- mot barn 15-17 år
- mot kvinna 18 år eller äldre
- mot man 18 år eller äldre

Övriga brott mot 3 kap.

4 kap. Brott mot frihet och frid

Människorov (1)

Människohandel (1a)

Olaga frihetsberövande (2)

därav grov fridskränkning (4a)

grov kvinnofridskränkning (4a)

olaga förföljelse (4b)

ofredande (7)

6 kap. Sexualbrott

1 § Våldtäkt

1 § Grov våldtäkt

2 § Sexuellt tvång

2 § Grovt sexuellt tvång

4 § Våldtäkt mot barn

4 § Grov våldtäkt mot barn

Delar av 8 kap. Stöld, rån m.m.

- Rån (inkl. grovt) (5§)
- därav bankrån (6§)

13 kap. Allmänfarliga brott

16 kap. Brott mot allmän ordning: 1-8 §

17 kap. Brott mot allmän verksamhet därav våld mot tjänsteman: §1-5 & 10
